# Genomic and Transcriptomic Profile of HNF1A-Mutated Liver Adenomas Highlights Molecular Signature and Potential Therapeutic Implications

**DOI:** 10.3390/ijms251910483

**Published:** 2024-09-29

**Authors:** Angelo Corso Faini, Francesca Arruga, Michele Pinon, Valeria Bracciamà, Francesco Edoardo Vallone, Fiorenza Mioli, Monica Sorbini, Martina Migliorero, Alessandro Gambella, Damiano Carota, Isaac Giraudo, Paola Cassoni, Silvia Catalano, Renato Romagnoli, Antonio Amoroso, Pier Luigi Calvo, Tiziana Vaisitti, Silvia Deaglio

**Affiliations:** 1 Immunogenetics and Transplant Biology Service, Azienda Ospedaliera-Universitaria Città della Salute e della Scienza, 10126 Turin, Italy; francesca.arruga@unito.it (F.A.); valeria.bracciama@unito.it (V.B.); fiorenza.mioli@unito.it (F.M.); monica.sorbini@unito.it (M.S.); antonio.amoroso@unito.it (A.A.); tiziana.vaisitti@unito.it (T.V.); silvia.deaglio@unito.it (S.D.); 2 Department of Medical Sciences, University of Turin, 10126 Turin, Italy; edoardo.vallone454@edu.unito.it (F.E.V.); martina.migliorero@unito.it (M.M.); 3 Pediatric Gastroenterology Unit, Regina Margherita Children’s Hospital, Azienda Ospedaliera-Universitaria Città della Salute e della Scienza, 10126 Turin, Italy; michele.pinon@gmail.com (M.P.); isaac.giraudo@gmail.com (I.G.); pierluigi.calvo@unito.it (P.L.C.); 4 Pathology Unit, Department of Medical Sciences, University of Turin, 10126 Turin, Italy; alessandro.gambella@unito.it (A.G.); damiano.carota@unito.it (D.C.); paola.cassoni@unito.it (P.C.); 5 Division of Liver and Transplant Pathology, University of Pittsburgh, Pittsburgh, PA 15260, USA; 6 General Surgery 2U, Liver Transplantation Center, Azienda Ospedaliera-Universitaria Città della Salute e della Scienza, 10126 Turin, Italy; silvia.catalano@unito.it (S.C.); renato.romagnoli@unito.it (R.R.)

**Keywords:** *HNF1A*, hepatic adenomas, liver, molecular signature, genetics

## Abstract

Hepatocellular adenomas (HAs) are tumors that can develop under different conditions, including in patients harboring a germline mutation in *HNF1A*. However, little is known about the pathogenesis of such disease. This work aims to better define what mechanisms lie under the development of this condition. Six HAs were sampled from the liver of a 17-year-old male affected by diabetes and multiple hepatic adenomatosis harboring the heterozygous pathogenic germline variant c.815G>A, p.(Arg272His) in *HNF1A,* which has a dominant negative effect. All HAs were molecularly characterized. Four of them were shown to harbor a second somatic *HNF1A* variant and one had a mutation in the *ARID1A* gene, while no additional somatic changes were found in the remaining HA and normal parenchyma. A transcriptomic profile of the same HA samples was also performed. *HNF1A* biallelic mutations were associated with the up-regulation of several pathways including the tricarboxylic acid cycle, the metabolism of fatty acids, and mTOR signaling while angiogenesis, endothelial and vascular proliferation, cell migration/adhesion, and immune response were down-regulated. Contrariwise, in the tumor harboring the *ARID1A* variant, angiogenesis was up-modulated while fatty acid metabolism was down-modulated. Histological analyses confirmed the molecular data. Independently of the second mutation, energetic processes and cholesterol metabolism were up-modulated, while the immune response was down-modulated. This work provides a complete molecular signature of *HNF1A*-associated HAs, analyzing the association between specific *HNF1A* variants and the development of HA while identifying potential new therapeutic targets for non-surgical treatment.

## 1. Introduction

*HNF1A* encodes a transcription factor binding to the 5′-GTTAATNATTAAC-3′ DNA sequence and is expressed in tissues of endodermal origin. It works as a dimer and interacts with other proteins such as CREB, EP300, PCAF, RAC3, Src, and HNF4A [1,2,3,4]. *HNF1A* maps on chromosome 12q24.2 and encompasses ten exons, which results in eight alternative isoforms. The protein contains three functional domains: an N-terminal dimerization domain, a POU homeodomain, and a C-terminal transactivation domain.

HNF-1α is involved in the transcriptional regulation of many different cell processes, particularly in the pancreas and liver. In the pancreas, HNF-1α is vital for the development and function of beta cells by regulating the expression of the insulin (*INS*), glucokinase (*GCK*), and glucose transporter (such as *GLUT2*) genes [5]. HNF-1α also plays a fundamental role in alpha cells by contributing to the balanced production of glucagon [6].

In the liver, HNF-1α regulates the expression of genes involved in glucose and lipid metabolism, albumin and bile acid synthesis, inflammation, drug metabolism, and detoxification processes. Among the most relevant targets are glucose-6-phosphatase, apolipoproteins, fibrinogen-alpha and -beta, albumin, alpha-fetoprotein, alpha-1-antitrypsin, liver-type pyruvate kinase, transthyretin, aldolase B, and hepatitis B virus large surface protein [7].

Pathogenetic variants of *HNF1A* have been associated with a spectrum of conditions that affect internal organs, particularly the liver and pancreas. Being a key regulator of sugar metabolism, it was initially defined as involved in the development of diabetes, particularly maturity-onset diabetes of the youth type III (MODY3, OMIM#600496) and diabetes mellitus, insulin-dependent type 20 (OMIM#612520). In addition to these two conditions, *HNF1A* variants have been described as susceptibility factors to other types of diabetes (OMIM#222100 and #125853) [8].

*HNF1A* pathogenic variants can also be responsible for liver adenomatosis (OMIM#142330), a benign condition characterized by multiple HAs in the liver of young patients [9,10]. Such a condition differs from the more common HAs of adults, which usually occur in young women taking oral contraceptives or corticosteroids. *HNF1A*-associated adenomatosis, although benign, is not easy to manage clinically and can lead to liver resections or even transplantation due to massive organ enlargement.

Based on this evidence, it was proposed that *HNF1A* acts as a tumor suppressor whose biallelic inactivation can be responsible for the onset of liver tumors, phenotypically characterized by a marked steatosis [10]. Animal models have also confirmed this hypothesis, showing that mice harboring homozygous or compound heterozygous variants in *HNF1A* develop liver enlargement associated with hepatocyte proliferation [11,12]. While some hypotheses have been proposed, the mechanisms through which *HNF1A* inactivation leads to HA formation remain incompletely understood.

The aim of the present work is to better define the role played by different *HNF1A* variants during HA transformation and the biological pathways that are dysregulated in this context to identify potential new pathogenic mechanisms that may be translationally exploited. To this end, six HAs from the liver of a 17-year-old male affected by diabetes and hepatic adenomatosis were sampled and profiled from the genomic and transcriptomic standpoints to obtain a more complete molecular characterization of this disease.

## 2. Results

### 2.1. Hepatic Adenomas Sampling

**Figure 1:**
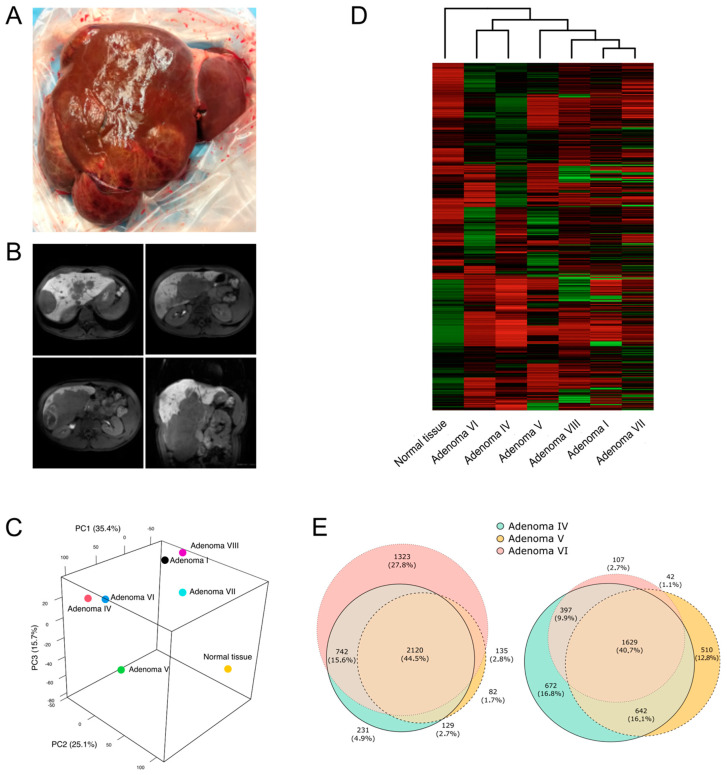
Morphological features of adenomas and molecular similarities between HAs. Picture (**A**) and representative transversal and coronal NMR scans (**B**) of the native liver of the patient. Axial T1-weighted gradient recall echo sequences with the phase opposition technique, single-shot T2 sequences with the fat suppression technique, and single-shot T2-weighted and diffusion echo-planar imaging sequences were acquired, and a dynamic study was performed with an intravenous infusion of hepatobiliary paramagnetic contrast medium (Gd-EOB-DTPA). (**C**) Principal component analysis (PCA) showing the HA samples clustering. (**D**) Heatmap showing differential gene expression profiles in each sample, reflecting PCA clustering. Hierarchical clustering shows that all HAs cluster together as opposed to normal parenchyma, which stands alone. Among the HA samples, adenomas IV and VI form a cluster as do adenomas I, VII, and VIII, while adenoma V seems to be a cluster on its own. (**E**) Venn diagrams showing the number and percentage of genes commonly up- or down-modulated (**left** and **right** panel, respectively) in adenomas IV, V, and VI. Each circle corresponds to the up- or down-modulated genes in each sample as compared to normal parenchyma.

The patient was a 17-year-old male affected by maturity-onset diabetes of the young (MODY) and treated with metformin (500 mg twice a day) who developed hepatic adenomatosis. No other significant diseases or events were reported in the patient’s clinical history. Upon referral to our center, he presented with multiple HAs with rapidly increasing volumes and necrotic and inflammatory evolution in segments I, IV, V, VI, VII, and VIII (Figure 1A). Representative NMR scans of the patient’s liver are shown in Figure 1B. Axial T1-weighted gradient recall echo sequences with the phase opposition technique, single-shot T2 sequences with the fat suppression technique, and single-shot T2-weighted and diffusion echo-planar imaging sequences were acquired, and a dynamic study was performed with an intravenous infusion of hepatobiliary paramagnetic contrast medium (Gd-EOB-DTPA). Necrotic areas are documented in segments VI, VII, and VIII, while the most caudal lesion appears exophytic with intralesional inflammatory characteristics.

Family history was informative as one brother, the mother, and an aunt from the maternal side presented with MODY. Additionally, another case of adenomatosis was reported in a second-grade maternal cousin. No genetic reports were available for any of the family members. On the paternal side of the family, no significant history of diabetes or hepatopathies was reported.

Following a multidisciplinary assessment, the patient underwent hepatectomy and orthotopic liver transplantation (OLT). Upon the removal of the native liver, six lesions and the corresponding healthy parenchyma were sampled. DNA and RNA were extracted from each tissue sample, and histologic and immunohistochemical analyses were performed.

OLT was successful and the patient recovered with no major complications. Of note, one month after the procedure, Epstein–Barr virus (EBV) reactivation was observed with no dangerous consequences and in March 2021, an acute rejection episode occurred, which was treated with high-dose steroids. As of now, the patient receives immunosuppressive therapy with tacrolimus + everolimus and is in good health condition with a fully functioning graft.

### 2.2. Adenoma Samples Showed the Presence of a Second Somatic Variant

CES performed on the patient’s peripheral blood mononuclear cell-derived DNA identified the germline c.815G>A, p.(Arg272His) [chr12:121432068G>A] pathogenic variant in the *HNF1A* gene in a heterozygous state. This variant was previously reported in association with maturity-onset diabetes of the young type 3 (MODY3), with a demonstrated dominant negative effect [13].

CES was also performed on samples from each HA, and data were aligned for somatic variant calling. Primary analysis was focused on the identification of additional variants in the *HNF1A* gene. In case of a negative result, the analysis was expanded to include a panel of 154 hepatic tumor-associated genes (Supplementary Table S1).

Non-germline and lesion-specific variants were filtered in, and each variant was singularly evaluated based on multiple parameters, including variant type, GnomAD frequency, nucleotide conservation, protein and splicing impact, and mapping quality.

In adenoma IV, a large likely pathogenic deletion, encompassing exons 3–10 of *HNF1A,* was identified. Lesion V was characterized by the presence of the pathogenic c.3575_3599del, p.(Asn1192Argfs*6) variant in the *ARID1A* gene, causing a frameshift and the generation of a premature stop codon with six amino acids downstream of asparagine 1192. In HA VI, the frameshift c.406dup, p.(Thr136Asnfs*52) variant in *HNF1A* was identified and was classified as likely pathogenic, generating a premature stop codon with 52 amino acids downstream of threonine 136. HA VII presented a likely pathogenic frameshift variant in *HNF1A* [c.1226del, p.(Pro409Leufs*4)] as well, causing the formation of a premature stop codon four amino acids downstream of proline 409. In HA VIII, a 12-nucleotide deletion was identified (c.714-1_724del), causing the loss of an intron–exon junction with a highly likely predicted impact on mRNA splicing and protein transduction (Table 1).

The germline and somatic variants were validated with alternative approaches (Supplementary Figure S1).

### 2.3. HNF1A Genetic Variants in Adenomas Control Different Molecular Pathways

To investigate the effects induced by the additional somatic mutations on the pathogenesis of HAs, RNA sequencing was performed for each tissue sample. A principal component analysis was performed (Figure 1C), and a hierarchically clustered heatmap was generated (Figure 1D). The results showed clustering of the HAs derived from segments IV and VI and of those derived from segments I, VII, and VIII. Normal parenchyma and the HA sampled from segment V appeared to stand alone and differ from all the others.

To better interpret these data, we looked at the NMR and histological features of these HAs, which were compatible with suboptimal sampling of the HAs and possibly with the presence of necrotic tissue within the biopsy. Considering this evidence, we decided to perform the following analyses considering only samples IV, V, and VI, in which RNA quality was acceptable and somatic mutations in *HNF1A* and *ARID1A* had been confirmed with Sanger sequencing and MLPA (Supplementary Figure S1).

For this reason, PCA was repeated in samples IV, V, and VI, and a DGE analysis of the first 30 genes modulated in components 1 and 2 was performed (Supplementary Figure S3). Genes clustered in the first principal component pertained to pathways involved in lipogenesis, fatty acid and cholesterol metabolism, oxidative phosphorylation, glycolysis, and DNA repair. In the second component allograft rejection, immune response and cell signaling, including mTOR and apoptosis, were the most significantly modulated pathways.

We next considered adenomas IV and VI together, both sharing a second mutation in *HNF1A* and clustering close with one another in the PCA and the hierarchical clustering. Genes commonly modulated in HAs IV and VI were compared to those selectively modulated in adenoma V, which presented a second hit in *ARID1A* (Figure 1E).

**Figure 2:**
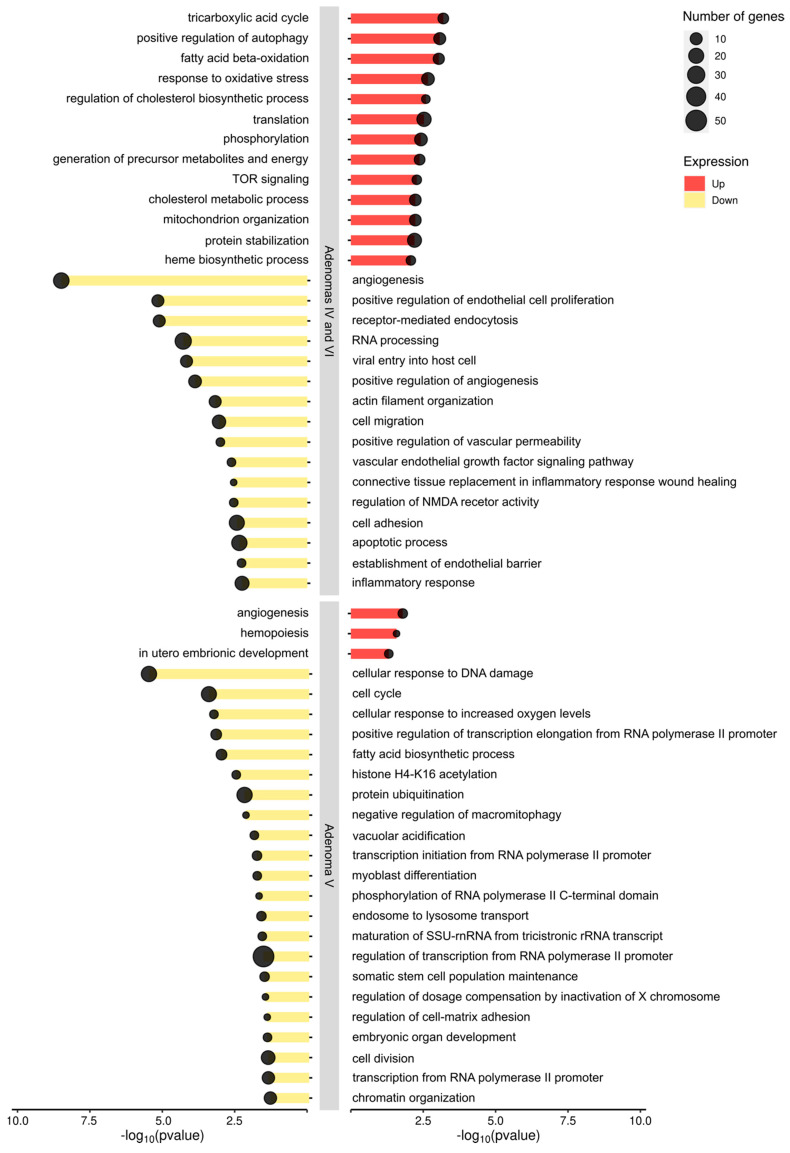
Graphical illustration of the commonly up- and down-modulated pathways in adenomas IV, VI, and V. Each modulated pathway is represented by a line whose length is proportional to the −log10(*p*-value). Red lines represent up-modulated pathways; yellow lines represent down-modulated pathways. Dot diameter is proportional to the number of genes modulated in each pathway.

Differential gene expression (DGE) analysis showed the up-modulation of several pathways, including tricarboxylic acid cycle, fatty acid beta-oxidation, and mTOR signaling. A down-modulation of the pathways involved in angiogenesis, endothelial proliferation, cell migration, adhesion, and vascular proliferation was observed in adenomas IV and VI. Contrariwise, in adenoma V, angiogenesis and hemopoiesis appeared selectively up-modulated, while cellular response to DNA damage, cell cycle, fatty acid biosynthesis, and RNA processing were down-modulated (Figure 2).

**Figure 3:**
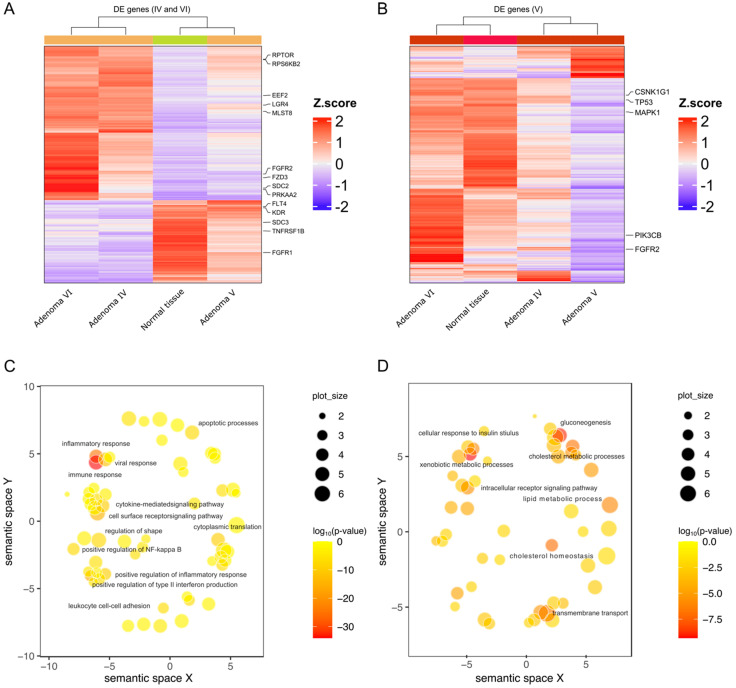
Differential gene expression in adenomas IV, V, and VI. Heatmaps representing modulated pathways in adenomas IV, VI (**A**), and V (**B**) compared to normal tissue. Modulated genes that are involved in the mTOR signaling pathway are reported on the right side of each graph. (**C**,**D**) Scatter plots representing the most significantly down- (**C**) and up- (**D**) modulated pathways in all three studied adenomas (IV, V, and VI, independently of the somatic mutation). Colors represent −log10(*p*-value) and the dot diameter is proportional to the number of genes modulated in each pathway. Immune response and inflammation appear down-regulated, while energetic processes and cholesterol metabolism are up-modulated.

Among these pathways, mTOR signaling has already been described in the literature as possibly involved in adenoma genesis. For this reason, we assessed whether there were genes taking part in the mTOR signaling among those selectively modulated in adenomas IV, V, and VI. The results of this analysis showed that in adenoma IV, fourteen genes belonging to the mTOR pathway were present, while only five were identified in adenoma V (Figure 3A,B).

We also wondered if, independently of the second mutation, some specific pathways were modulated. To address this point, GO analysis was performed on the genes commonly up- and down-modulated in adenomas IV, V, and VI. The results highlighted that immune response, inflammation, and interferon production were significantly down-modulated while gluconeogenesis, xenobiotic metabolic processes, and cholesterol homeostasis were up-modulated (Figure 3C,D), which is consistent with a tumor phenotype characterized by immune modulation and high energetic activity.

**Figure 4:**
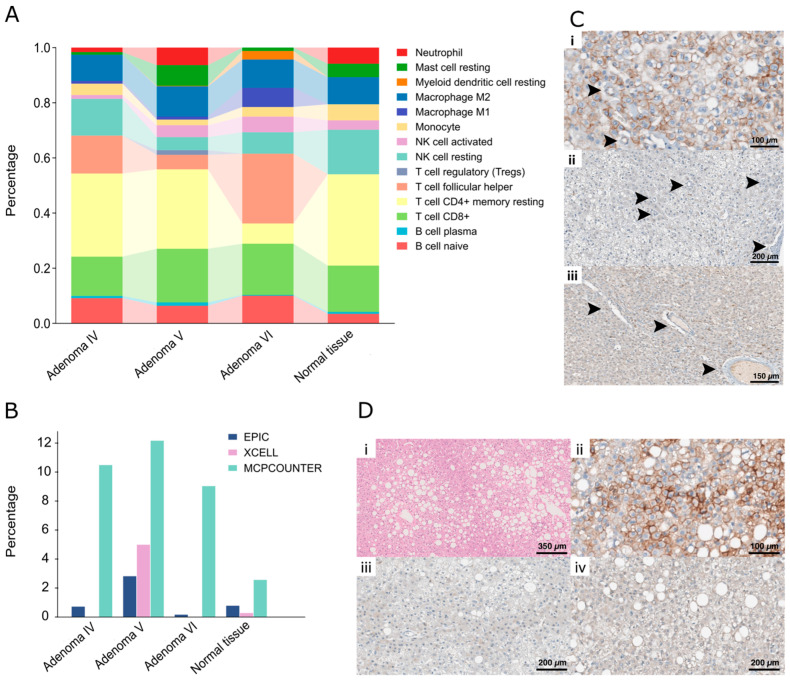
Cell population analysis and morphological and immunohistochemical profile of the HA. (**A**) Bar plot showing the cellular composition of HA IV, V, VI, and normal tissue. HAs, particularly adenoma VI, seem to be enriched in follicular T-helper cells, which are not present in normal tissue. On the other hand, adenoma VI seems to have a lower number of resting CD4^+^ T memory cells and a higher percentage of resting dendritic cells. Of note, adenoma V has a small amount of T regulatory cells, which are not present in the other samples. Also, adenoma V is richer in resting mast cells as compared to the other adenomas. (**B**) Graph showing the percentage of endothelial cells in each studied HA. Three different algorithms were used and all of them—particularly EPIC and EXCELL—showing that adenoma V is richer in endothelial cells. (**C**) Upon histological analysis, adenoma V presented a wild-type IHC staining pattern for beta-catenin ((**Ci**) 400× original magnification), negative IHC stain for glutamine ((**Cii**) 200× original magnification), and negative HNF-1α expression in the nuclei of neoplastic hepatocytes ((**Ciii**) 400× original magnification). (**D**) The steatotic lesions presented diffuse large droplet macrovesicular steatosis involving approximately 40% of neoplastic cells ((**Di**) HE, 100× original magnification). IHC for beta-catenin showed a wild-type staining pattern in neoplastic hepatocytes, characterized by negative nuclear and positive membranous staining ((**Dii**) 400× original magnification). IHC for glutamine synthetase was negative in all neoplastic hepatocytes ((**Diii**) 200× original magnification). HNF-1α ((**Div**) 200× original magnification) was negative in neoplastic hepatocyte nuclei. Remarkably, the lesion of segment V presented less prominent steatosis (involving 20% of neoplastic cells, approximatively) but a slightly increased density of aberrant vessels (black arrowheads, (**Ci**–**Ciii**)).

Next, analyses of cell populations were performed using the CIBERSORT model, which is considered one of the most accurate [14]. Using this approach, all HAs, particularly adenoma VI, appeared richer in follicular T-helper cells, which were absent in normal tissue. On the other hand, adenoma VI showed a lower number of resting CD4^+^ T memory cells and a higher percentage of resting dendritic cells. Of note, adenoma V presented a small amount of T regulatory cells, which were apparently absent in the other samples. Also, adenoma V is enriched in resting mast cells as compared to the other HAs (Figure 4A). However, considered together, these changes in cell composition appear modest and unable to account for the differences observed by GO analysis.

Considering that the CIBERSORT model does not provide an estimate of the endothelial cell number, which we speculated could be increased in adenoma V, the same analysis was performed using the MPCOUNTER model, which includes this cell type. The results indicate that particularly adenoma V, when compared to normal tissue and to the other HAs, had a higher number of endothelial cells, which is consistent with the observation that in this HA, angiogenesis appeared up-modulated (Figure 4B).

### 2.4. Histopathological Analyses Reflect the Molecular Characteristics of Each Adenoma

To confirm findings obtained by analyzing RNA sequencing data, histopathological evaluation of samples obtained from the HAs was performed. Morphological characteristics of the lesions were analyzed, as well as HA tumor cell beta-catenin, glutamine synthetase, and HNF-1α protein expression via immunohistochemistry (IHC).

Consistent with the results obtained from RNA sequencing, the lesion of segment V presented an increased density of aberrant vessels (black arrowheads, Figure 4C) and less prominent steatosis (involving 20% of neoplastic cells, approximately). Beta-catenin staining resulted in a wild-type pattern, while glutamine synthase and HNF-1α were negative (Figure 4(Ci–Ciii)).

The steatotic lesions sampled from hepatic segments IV and VI presented diffuse large droplet macrovesicular steatosis involving ≅40% of neoplastic cells (Figure 4(Di)). IHC for beta-catenin showed a wild-type staining pattern of neoplastic hepatocytes in all analyzed HAs, characterized by negative nuclear and positive membranous staining (Figure 4(Dii)). Glutamine synthetase was negative in all neoplastic hepatocytes, and HNF-1α was negative in neoplastic hepatocyte nuclei (Figure 4(Diii,Div)).

## 3. Discussion

Although rare and benign, liver adenomatosis can become a potentially life-threatening situation, as liver architecture and function can be deeply affected. The most recent evidence on the pathogenesis of HAs reports several different causes, among which is the postulated biallelic loss of function (LOF) of *HNF1A* [15,16,17]. However, little is known about the molecular mechanisms that lead HNF-1α -deficient hepatocytes to evolve into HAs.

Here, we provide molecular characterization of different HAs obtained from the same patient, describing the effects of biallelic inactivation of *HNF1A*, as well as its role as a tumor suppressor. To this aim, we molecularly profiled six HAs from a patient harboring a germline mutation in *HNF1A* with a negative dominant effect [18] who developed massive hepatic adenomatosis. Overall, our results reveal that in the majority of the sampled HAs, *HNF1A* presents a second LOF mutation. However, considering that not all patients harboring pathogenic variants in *HNF1A* develop liver adenomatosis, one may speculate that only certain germline variants induce adenomatous transformation. In support of this hypothesis, almost all reported cases of HNF-1α-inactivated adenomatosis are due to LOF germline variants that are reasonably followed by a somatic stochastic second hit on the other *HNF1A* allele [16,19,20,21,22,23]. Of note, other cases of adenomatosis in patients harboring the same germline variant as our patient are reported [24,25], suggesting that this particular variant generates a non-functional protein with loss of DNA binding activity and interaction with other proteins [13].

To better define what pathways are dysregulated upon *HNF1A*-dependent adenomatous transformation, we analyzed the transcriptional profile of the different HAs. Among the up-regulated pathways, the tricarboxylic acid cycle, metabolism of fatty acids, and mTOR signaling were prominent, confirming previous works [26,27].

In addition to these, other pathways were up-modulated, such as autophagy and response to oxidative stress. On the other hand, angiogenesis, endothelial and vascular proliferation, cell migration and adhesion, and immune response were markedly down-regulated, coherent with a benign behavior of HAs while partially questioning previous evidence in which angiogenesis was hypothesized to be up-regulated [26].

However, the picture appeared more complicated, as we observed that a second somatic mutation involving genes other than *HNF1A* may also likely trigger transformation and even have a different impact on the phenotypic characteristics of the HA, as different subtypes of HAs, as well as malignant transformation, are reported [28,29,30,31]. In fact, one specific HA, harboring a second mutation in *ARID1A*, presented a distinct molecular signature with an up-regulation of angiogenesis and a down-regulation of cellular response to DNA damage, cell cycle, fatty acid biosynthesis, and RNA transcription and processing, which were previously speculated to be up-regulated [26]. This evidence was consistent with histological analysis, which showed different characteristics of this peculiar HA. A marked steatosis of inactivated HNF-1α HAs was in fact substituted by an enriched vascularity and an apparently more aggressive phenotype in the *ARID1A*-mutated HA, which is consistent with reports of malignant transformation of *HNF1A*-related HAs [32].

Independently of the second mutation, we observed that energetic processes and cholesterol metabolism were up-modulated. This could be at least partly explained by the presence of a germline dominant negative mutation leading to an HNF-1α-mediated down-regulation of *PCSK9* transcription and subsequent decreased LDLR degradation [33]. In fact, *HNF1A* inactivation impairs *PCSK9* transcription, thus preventing the degradation of LDLR molecules, which favors the internalization and metabolization of cholesterol in hepatocytes [34].

On the other hand, in all HAs, the immune response appeared down-regulated, which is consistent with the immunotolerant microenvironment observed in tumors. However, recent evidence on the pro-inflammatory activity of HNF-1α in the context of other hepatic diseases [35,36] suggests that further studies in hepatocytes and immune cells are needed to understand the role of *HNF1A* in the immune response.

Overall, our results identify a high-risk non-truncating *HNF1A* germline variant leading to early-onset multiple HAs, providing their extensive molecular characterization and defining dysregulated pathways and somatic variants occurring upon hepatocyte transformation. Also, our work highlights the importance of a correct definition not only of the histological features but also of the genetic characteristics and background of HAs, which could be helpful in determining their malignant transformation potential and in stratifying risk. Moreover, these observations might be relevant as the definition of a more complete molecular signature of HAs can help in identifying potential new targets exploitable in the context of non-surgical therapy of adenomatosis. Such disease is in fact often treated with liver resections or transplantation, which are major surgeries with possibly dangerous complications and a life-long need for immunosuppression. Our results showed the down-regulation of the mTOR pathway—as previously observed also in the presence of dominant negative variants [26,37]—could be identified as a potential therapeutic target for the pharmacological treatment of adenomatosis. Indeed, Rapamycin proved effective in mitigating pancreatic beta cell damage in *HNF1A*-associated diabetes [38], and it is worth considering the possibility of using such a compound also in a post-transplantation setting.

However, this study presents some limitations that must be acknowledged. The main element that can introduce bias comes from the fact that all adenomas were sampled from a single patient, which may indeed impact the diversity and representativeness of the collected data and limit the generalizability of the results. This is in part due to the rarity of the disease and to the difficulties encountered in sampling and obtaining material with sufficiently high quality for molecular analyses. A second aspect to be considered is that the tests performed are not exhaustive and—particularly in those samples in which no additional variants were found—some other phenomena contributing to the development of the adenoma may have occurred and not be detected at the genomic or transcriptomic level.

## 4. Materials and Methods

### 4.1. Sample Collection

All research was conducted in accordance with both the Declarations of Helsinki and Istanbul following institutional ethical approval (protocol No. 679265 dated 7 March 2022). The patient’s parents signed the informed consent.

Samples from 6 HAs and the corresponding healthy parenchyma were collected straight after hepatectomy for transplantation purposes. Samples were rapidly formalin-fixed and paraffin embedded. Samples used for genomic and transcriptomic analyses underwent DNA and RNA extraction (Cat. No. 69506 Qiagen, Hilden, Germany and Cat. Nos. 15596026 and 15596018, Invitrogen, Waltham, MA, USA, respectively) following the manufacturer’s protocols. The obtained DNA and RNA were checked for quality and stored at −20 °C and −80 °C, respectively.

### 4.2. Clinical Exome Sequencing (CES)

Libraries were prepared using the TruSight One Expanded Sequencing Kit (Illumina, San Diego, CA, USA) following the manufacturer’s instructions.

Raw data were converted to FASTQ files and aligned with Enrichment 3.1.0 or DRAGEN Enrichment tools (Illumina, San Diego, CA, USA ) using Homo Sapiens UCSC GRCh37 genome as a reference and allowing for either germline or somatic variant calling. Vcf files for single-nucleotide variants, copy number variants, and structural variants were obtained. Data analysis was performed with Illumina Variant Interpreter software (version 2.17.0.60, Illumina, San Diego, CA, USA), which is freely available online. Blood samples were analyzed with the germline analysis protocol, while hepatic samples were analyzed with the somatic mutations protocol. When needed, variants were classified according to the American College of Medical Genetics and Genomics (ACMG) criteria.

A gene list of 154 hepatic tumor-associated genes was used to filter relevant variants (Supplementary Table S1). Gene selection was performed based on the literature, OMIM (https://www.omim.org, accessed on 13 April 2022), The Cancer Genome Atlas (https://www.cancer.gov/ccg/research/genome-sequencing/tcga, accessed on 13 April 2022), Cosmic (https://cancer.sanger.ac.uk/cosmic, accessed on 13 April 2022), and Cbio (https://www.cbioportal.org, accessed on 22 April 2022). For the latter two databases, only genes found mutated in >5% of the available samples were included.

### 4.3. Sanger Sequencing

Sanger sequencing of selected variants identified by NGS was performed using standard methods, as reported in [39]. Detailed primer sequences are reported in Supplementary Table S2. Sanger reaction was performed using the ProDye Terminator Sequencing System kit (Cat. No. CR4324, Promega, Madison, WN, USA) and sequencing on ABI 3100 Genetic Analyzer (Applied Biosystems, Foster City, CA, USA). Electropherograms were analyzed using the Chromas software (version 2.6, Technelysium, South Brisbane, Australia), which is freely available at www.technelysium.com.au.

### 4.4. Multiplex Ligation-Dependent Probe Amplification (MLPA)

MLPA was performed using the SALSA MLPA Probemix P241 MODY Mix 1 kit (Cat. No. P241-025R MRC Holland, Amsterdam, Netherlands) following the manufacturer’s protocol. Sequencing was performed on ABI 3100 Genetic Analyzer (Applied Biosystems, Foster City, CA, USA), and data wer analyzed using Coffalyzer software (version 9.4, MRC Holland, Amsterdam, Netherlands).

### 4.5. Digital Droplet PCR

A droplet digital PCR reaction mixture included 5ng of input DNA, 11 μL of 2x QX200 ddPCR EvaGreen Supermix, primers 10 nM, and 8.1 μL of water, for a final volume of 22 μL. Twenty μL of the sample were loaded on the QX100 Droplet Generator (Bio-Rad, Hercules, CA, USA) together with 70 μL of Droplet Generation Oil for EvaGreen (Cat. No. 1864005, Bio-Rad, Hercules, CA, USA). Forty μL of generated droplets were amplified in a T100 Thermal Cycler (Bio-Rad, Hercules, CA, USA). The thermal cycling conditions were 95 °C, 5 min; 95 °C, 40 cycles (95 °C, 30 s; 62 °C, 1 min); 4 °C, 5 min; 90 °C, 5 min; and 4 °C, hold. Fluorescence for each assay was measured by the QX200 Droplet Reader, and results were analyzed using QX manager 1.2 Standard edition (Bio-Rad, Hercules, CA, USA). The threshold was established based on the background of no template control (NTC) samples, whereas gDNA from a healthy donor’s PBMC was used as a positive control. All measurements were performed in triplicate. Primers used are reported in Supplementary Table S3.

### 4.6. RNA Sequencing

RNA quality was assessed by Bioanalyzer high-sensitivity RNA analysis (Cat. No. 5067-1511, Agilent, Santa Clara, CA, USA). Libraries were prepared using the Illumina Stranded Total RNA Prep with Ribo-Zero Plus (Cat. No. 20040525, Illumina, San Diego, CA, USA) following the manufacturer’s protocol.

The tidyverse R package v2.0.0 was used for the principal component analysis (PCA). Z-score-normalized TPMs were used for the analysis, plotting the first three components. Gene ontology (GO) analysis was performed with DAVID (version v2023q4, https://david.ncifcrf.gov/tools.jsp) [40,41] and Revigo (version 1.8.1, http://revigo.irb.hr) [42] online software. Graphical representations were performed with R (version 4.2.3 https://www.r-project.org/) and SRplot (https://www.bioinformatics.com.cn/srplot) software.

Additionally, the PCA of the TPMs of the differentially expressed genes (DEGs) was carried out with the mixOmics R package v6.26.0 [43]. Furthermore, GO analyses of the first 30 genes of each component (p1 and p2) were also performed with the enrichR R package v3.2 using the MSigDb Hallmark 2020 database [44].

Heatmaps were generated through the ComplexHeatmap R package v2.16.0., and Z-score-normalized TPMs were used as inputs [45,46]. K-means clustering was performed both on rows and columns. Data were plotted as −log10 of the adjusted *p*-values (or *p*-values, when no significant adj. *p*-value was present) using the ggplot2 R package v3.4.4 [47].

Cell subpopulation estimation analysis was performed using TIMER2.0 (version 2.0 http://timer.cistrome.org/) by considering the CIBERSORT [48], MCPCOUNTER [49], EPIC [50], and EXCELL [51] estimation scores.

### 4.7. Real-Time PCR

RNA from each sample was retrotranscribed using the high-capacity cDNA reverse transcription kit (Cat. No. 4368814, ThermoFisher Scientific, Waltham, MA, USA). Real-time PCRs were performed using the iTaq Universal Probes Supermix (Cat. No. 1725134, BioRad, Hercules, CA, USA), the CFX Opus 384 Real Time PCR system (BioRad, Hercules, CA, USA) and analyzed with CFX Maestro software (version 2.3, BioRad, Hercules, CA, USA). Assays for *FLT4* (Hs01047677_m1), *KDR* (Hs00911700_m1), *MTOR* (Hs00234508_m1), *NRP2* (Hs00187290_m1), *LARP1* (Hs00391726_m1), *EHHADH* (Hs00157347_m1), *MLST8* (Hs00909882_g1), *PALB2* (Hs00954121_m1), *ELOVL6* (Hs00907564_m1), *NOTCH4* (Hs00965889_m1), *TP53* (Hs01034249_m1), *TNFA* (Hs01113624_g1), *TGFB* (Hs00998133_m1), *ACTB* (Hs99999903_m1), and *HNF1A* (Hs00167041 and Hs01551745) were all from ThermoFisher Scientific.

Data were analyzed with the 2^−ΔΔCt^ method to calculate the relative expression of the gene under analysis. For each gene, expression levels were computed as the difference (ΔCt) between the target gene threshold cycle (Ct) and Actin Ct (Supplementary Figure S2).

### 4.8. Histopathological Analysis and Immunohistochemical Staining

Hematoxylin and eosin (HE) slides of all lesions were reviewed by an expert pathologist for tissue quality assessment and the selection of representative formalin-fixed paraffin-embedded tissue blocks. From each block, three 4 μm thick sections were stained for glutamine synthetase (mouse monoclonal, clone GS-6, catalog number: 389M-1; Cell Marque, Merck KGaA, Darmstadt, Germany), beta-catenin (mouse monoclonal, clone 14, catalog number: 224M-1; Cell Marque, Merck KGaA, Darmstadt, Germany ), and HNF-1α (rabbit polyclonal, Cat. No. ab96777, Abcam, Cambridge, UK). Immunohistochemical stains (IHCs) were performed on a Ventana BenchMark ULTRA AutoStainer (Ventana Medical Systems, Oro Valley, AZ, USA). Histopathological and immunohistochemical features were independently evaluated by two pathologists (AG and DC), and, in case of disagreement, the findings were discussed collectively to reach a consensus classification.

## 5. Conclusions

This work took advantage of both genomic and transcriptomic analyses of hepatic HNF1A-mutated adenoma samples to identify genotypic–phenotypic correlations and define a signature of dysregulated pathways and somatic variants occurring upon hepatocyte transformation. This could result in the identification of new therapeutic targets for the pharmacological treatment of liver adenomatosis.

## Figures and Tables

**Table 1:** A list of the somatic variants identified in each HA sample referred to the canonical NM_000545.8 and NM_006015.6 transcripts for *HNF1A* and *ARID1A*, respectively.

Sample	Gene	Additional Somatic Mutation	Chromosomic Position (hg37)	Variant Frequency	ACMG Classification
Normal parenchyma	/	/	/	/	/
Adenoma I	/	/	/	/	/
Adenoma IV	*HNF1A*	Deletion of exons 3–10	chr12:121431312-121613291del	0.05	C4
Adenoma V	*ARID1A*	c.3575_3599del, p.(Asn1192Argfs*6)	chr1:27099336	0.15	C4
Adenoma VI	*HNF1A*	c.406dup, p.(Thr136Asnfs*52)	chr12:121426714	0.35	C4
Adenoma VII	*HNF1A*	c.1226del, p.(Pro409Leufs*4)	chr12:121434460	0.16	C4
Adenoma VIII	*HNF1A*	c.714-1_724del	chr12:121431966-121431977	0.07	C4

## Data Availability

The raw datasets supporting the conclusions of this article are available online on the GEO database with accession number GSE268376. (https://www.ncbi.nlm.nih.gov/geo/query/acc.cgi?acc=GSE268376).

## Supplementary Materials

The following supporting information can be downloaded at https://www.mdpi.com/article/10.3390/ijms251910483/s1.
