# Supplementary material for: Genomic and Transcriptomic Profile of HNF1A-Mutated Liver Adenomas Highlights Molecular Signature and Potential Therapeutic Implications
Source: Int J Mol Sci. 2024 Sep 29;25(19):10483. doi: 10.3390/ijms251910483 (PMC11477380; doi:10.3390/ijms251910483)
Supplement: Supplementary file 1 [file ijms-25-10483-s001.zip › ijms-3206104-supplementary.pdf]

Supplementary Table S1 list of hepatic tumor-associated genes

154 liver tumor-associated genes						
ABCA13	*CACNA1A*	*DMD*	*HERC2*	*MAOB*	*PCLO*	*SLC8A1*
ACVR2A	*CACNA1B*	*DNAH5*	*HMCN1*	*MET*	*PDGFRL*	*SLCO1B3*
ADGRV1	*CACNA1C*	*DNAH7*	*HNF1A*	*MSH2*	*PIK3CA*	*SMARCA4*
AHNAK2	*CACNA1E*	*DNAH9*	*HNF4A*	*MSH6*	*PKHD1*	*SPEG*
ALB	*CAMTA1*	*DPP6*	*HRAS*	*MUC4*	*PKHD1L1*	*SPTA1*
ANK2	*CASP8*	*DSCAM*	*HYDIN*	*MUC5B*	*PLXNA4*	*SYNE1*
ANK3	*CCND1*	*DST*	*IGF2R*	*MYC*	*PMS2*	*SYNE2*
ANKS1B	*CCNE1*	*DYNC2H1*	*IL6R*	*MYO18B*	*PRKAR1A*	*TENM4*
APC	*CDH18*	*EYS*	*IL6ST*	*MYT1L*	*PTEN*	*TERT*
APOB	*CDKN1A*	*FANCA*	*IRF2*	*NBEA*	*PTPN13*	*THSD7B*
ARID1A	*CDKN2A*	*FAT3*	*JAK1*	*NEB*	*PTPRB*	*TP53*
ARID2	*CDKN2B*	*FAT4*	*KEAP1*	*NFE2L2*	*PTPRN2*	*TSC1*
ASH1L	*CHL1*	*FBN2*	*KIAA1217*	*NRAS*	*RB1*	*TSC2*
ATM	*COL11A1*	*FGF3*	*KMT2C*	*NRXN1*	*ROBO2*	*TSPEAR*
AXIN1	*COL12A1*	*FLG*	*KMT2D*	*NRXN3*	*RP1L1*	*TTN*
AXIN2	*CREB3L3*	*FMN2*	*KRAS*	*OBSCN*	*RPS6KA3*	*UBR3*
BAP1	*CSMD1*	*FRAS1*	*LAMA1*	*PCDH15*	*RYR1*	*UNC13C*
BRAF	*CSMD3*	*FREM1*	*LAMA2*	*PCDHA1*	*RYR2*	*USH2A*
BRCA1	*CTNNA2*	*FREM2*	*LRP1B*	*PCDHA10*	*RYR3*	*VPS13B*
BRCA2	*CTNNB1*	*FRK*	*LRP2*	*PCDHA13*	*SETD2*	*WDFY4*
BRIP1	*CUBN*	*GUCY1A2*	*LZTR1*	*PCDHA3*	*SETDB1*	*ZFPM2*
BUB1	*DCC*	*HERC1*	*MACF1*	*PCDHGB4*	*SF3B1*	*ZIC3*

Supplementary Table S2 list and sequence of primers used for Sanger sequencing

***ARID1A*_exon_14**	
FW	5’-GTGACTCCTGCGTGTCCTTT-3’
REV	5’-CCACAAGAACCCTGAGCCAT-3’
***HNF1A*_exon_2**	
FW	5’-CCCACCTATGGGGAGAGACA-3’
REV	5’-CATTACTTACGCTGCGCCAC-3’
***HNF1A*_exon_6**	
FW	5’-TTGGCTTCCCCTCGTAGGT-3’
REV	5’-GCTTACCGATGACCAGGGTG-3’

Supplementary Table S3: list and sequence of primers used for ddPCR

*HNF1A*_exon_4_mutated	
FW	5’-CTTCTCAGAACCCTCCCCTTC-3’
REV	5’-ACCCCTCTCTGGATGCAGAA-3’
***HNF1A*_exon_4_WT**	
FW	5’-CTTCTCAGAACCCTCCCCTTC-3’
REV	5’- TGCATTCCGCCCTGCA-3’

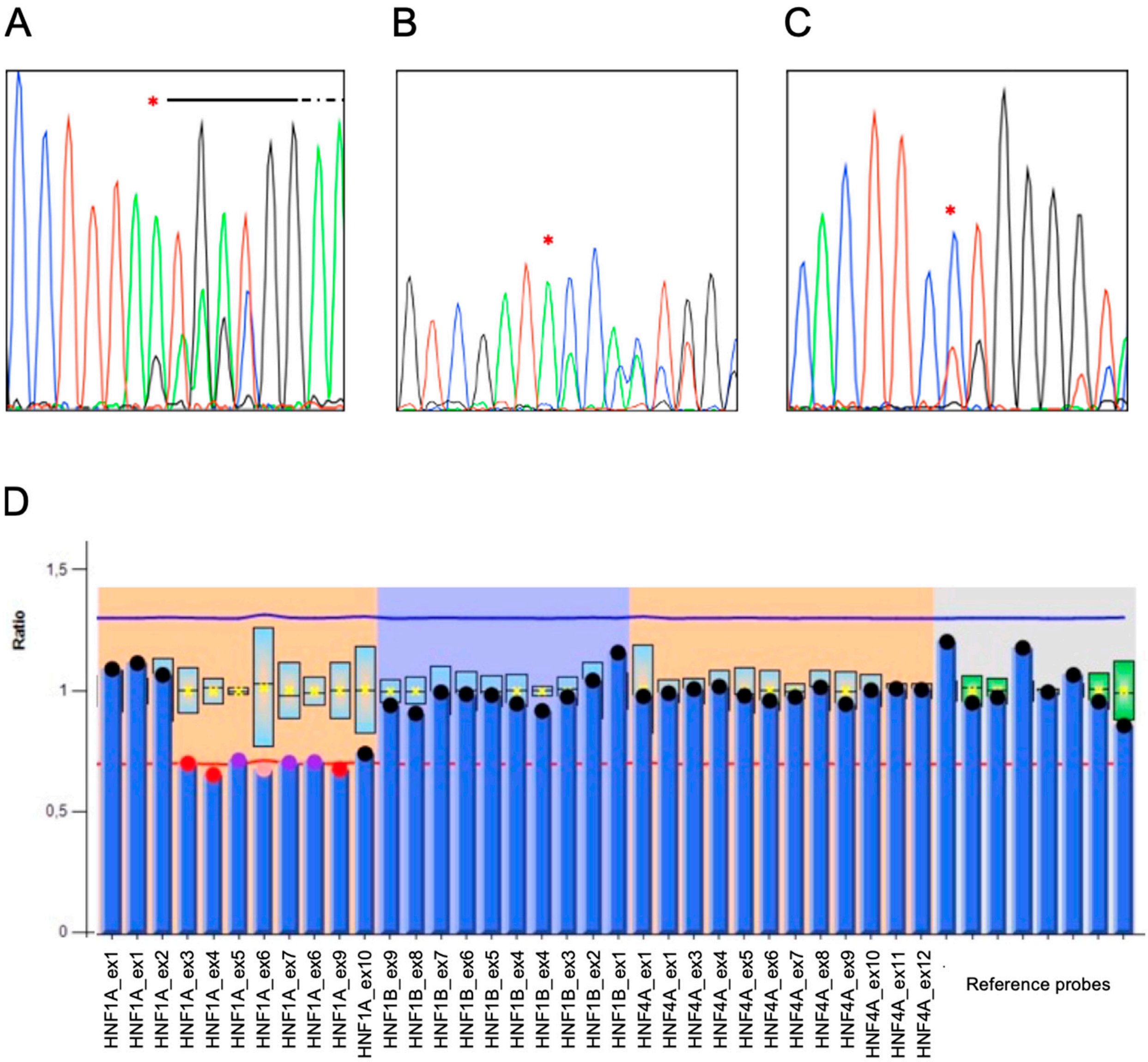

Supplementary Figure S1 Chromatograms showing Sanger sequencing validation of identified variants. *ARID1A* c.3575_3599del, p.(Asn1192Argfs*6) mutation on adenoma V (A), *HNF1A* c.406dup, p.(Thr136Asnfs*52) mutation on adenoma VI (B) and *HNF1A* c.1226del,

p.(Pro409Leufs*4) mutation on adenoma VII (C). Asterisks indicate the variant in the chromatograms. MLPA validation of the somatic *HNF1A* deletion (chr12:121431312-121613291) on adenoma IV (D). *HNF1A* and *HNF4A* genes were used as controls and resulted wild type with no deletions or duplications

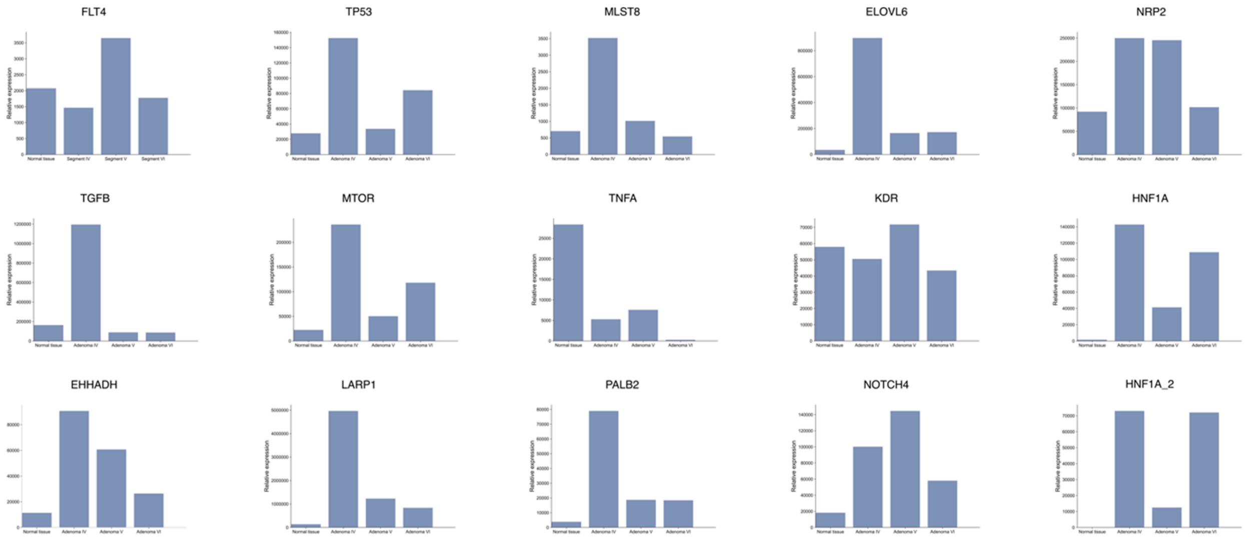

Supplementary Figure S2 Bar plots representing relative expression of selected genes obtained by RT-PCR. For each gene, expression levels were computed as the difference (ΔCt) between the target gene threshold cycle (Ct) and Actin Ct

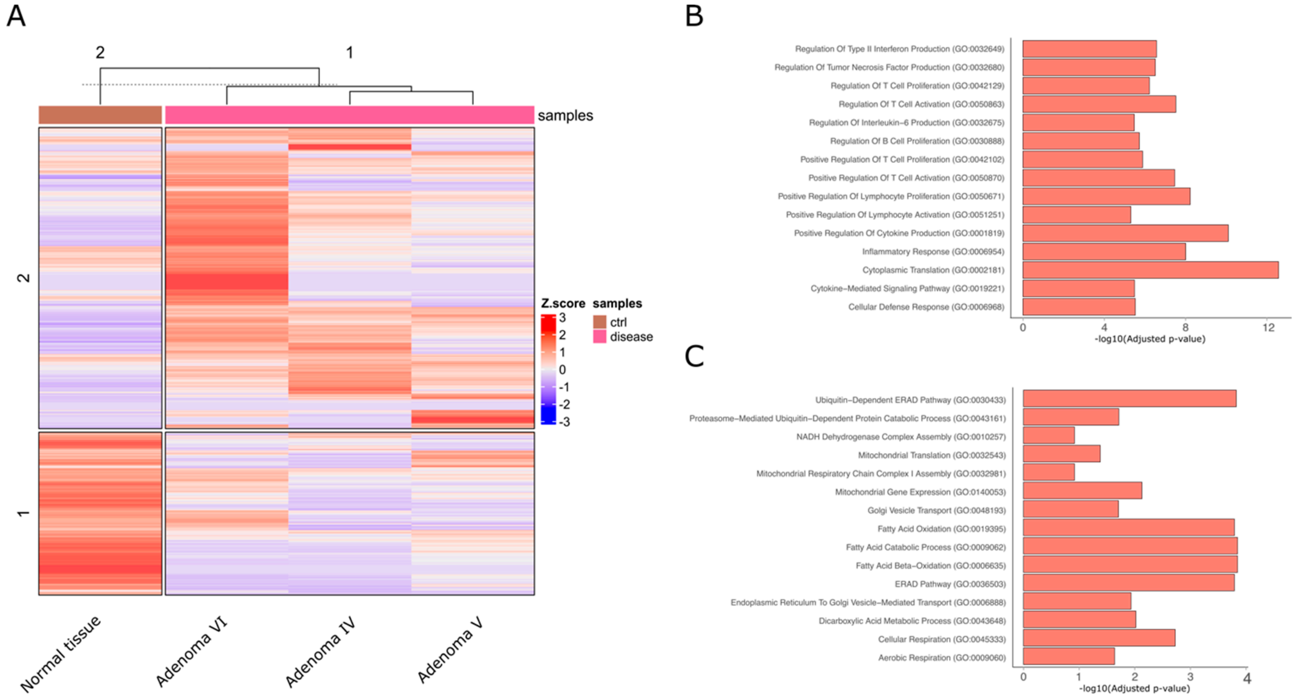

Supplementary Figure S3 hierarchical clustering of RNA sequencing data from segments IV, V and VI (A). The first 30 most significantly modulated genes in components 1 and 2 were used for the DGE analysis (B and C). Lipogenesis, fatty acid and cholesterol metabolism, oxidative phosphorylation, glycolysis and DNA repair were the most significantly modulated pathways in component 1 while in component 2 allograft rejection, immune response, cell signaling including mTOR and apoptosis were the most significantly modulated pathways
